# Are Epigenetic Factors Implicated in Chronic Widespread Pain?

**DOI:** 10.1371/journal.pone.0165548

**Published:** 2016-11-10

**Authors:** Andrea Burri, Zoya Marinova, Mark D. Robinson, Brigitte Kühnel, Melanie Waldenberger, Simone Wahl, Sonja Kunze, Christian Gieger, Gregory Livshits, Frances Williams

**Affiliations:** 1 Health and Rehabilitation Research Institute, Auckland University of Technology, Auckland, New Zealand; 2 Waitemata Pain Service, Department of Anaesthesia and Perioperative Medicine, North Shore Hospital, Auckland, New Zealand; 3 Department of Psychology, University of Zurich, Binzmühlestrasse 14, 8050 Zurich, Switzerland; 4 Department of Psychosomatic Medicine, Clinic Barmelweid, Barmelweid 5017, Switzerland; 5 SIB Swiss Institute of Bioinformatics, University of Zurich, 8057 Zurich, Switzerland; 6 Institute of Molecular Life Sciences, University of Zurich, 8057 Zurich, Switzerland; 7 Research Unit of Molecular Epidemiology and Institute of Epidemiology II, Helmholtz Zentrum München, Munich, Germany; 8 Sackler Faculty of Medicine, Tel Aviv University, Tel Aviv, Israel; 9 Department of Twin Research and Genetic Epidemiology, King’s College London, St.Thomas´ Hospital, Westminster Bridge Road SE1 7EH, London, United Kingdom; Boston Children’s Hospital and Harvard Medical School, UNITED STATES

## Abstract

**Background:**

Chronic widespread musculoskeletal pain (CWP) is the cardinal symptom of fibromyalgia and affects about 12% of the general population. Familial aggregation of CWP has been repeatedly demonstrated with estimated heritabilities of around 50%, indicating a genetic susceptibility. The objective of the study was to explore genome-wide disease-differentially methylated positions (DMPs) for chronic widespread pain (CWP) in a sample of unrelated individuals and a subsample of discordant monozygotic (MZ) twins.

**Methodology/Principle Findings:**

A total of N = 281 twin individuals from the TwinsUK registry, including N = 33 MZ twins discordant for self-reported CWP, were part of the discovery sample. The replication sample included 729 men and 756 women from a subsample of the KORA S4 survey–an independent population-based cohort from Southern Germany. Epigenome-wide analysis of DNA methylation was conducted using the Illumina Infinium HumanMethylation 450 DNA BeadChip in both the discovery and replication sample. Of our 40 main loci that were carried forward for replication, three CPGs reached significant p-values in the replication sample, including malate dehydrogenase 2 (*MDH2*; p-value 0.017), tetranectin (*CLEC3B*; p-value 0.039), and heat shock protein beta-6 (*HSPB6*; p-value 0.016). The associations between the collagen type I, alpha 2 chain (*COL1A2*) and monoamine oxidase B (*MAOB)* observed in the discovery sample–both of which have been previously reported to be biological candidates for pain–could not be replicated.

**Conclusion/Significance:**

Our results may serve as a starting point to encourage further investigation in large and independent population-based cohorts of DNA methylation and other epigenetic changes as possible disease mechanisms in CWP. Ultimately, understanding the key mechanisms underlying CWP may lead to new treatments and inform clinical practice.

## Introduction

Chronic widespread musculoskeletal pain (CWP) is the cardinal symptom of fibromyalgia (FM), emphasizing axial pain as a constant feature, as well as the presence of pain in the upper and lower quadrants of abdomen, and the right and left sides of the body [1]. Musculoskeletal pain affects about 12% of the general population, with the proportion of women being generally twice as high compared to men [2]. CWP is a diagnosis that challenges the social and cultural boundaries of current perception of illness and health in the Western world and the lack of a biomedical diagnosis denies the sufferer's legitimacy of the "sick role". CWP is not only a huge burden for sufferers, but also difficult to treat and costly for society overall. Despite recent research efforts, the CWP etiology and mechanisms remain unclear, with evidence from a range of epidemiologic studies suggesting an interplay of socio- demographic, psychosocial, affective, physiological, as well as genetic factors [3–8].

Familial aggregation of CWP has been repeatedly demonstrated with estimated heritabilities of around 50% [3,5]. Although several studies have been performed to search for biomarkers, they have been mostly underpowered, restricted by the heterogeneity of the phenotype, constrained by study design, and consequently remain unreplicated [9]. Nevertheless, several biochemical factors (e.g., androgens) [10] and candidate genes—in particular genes involved in neurotransmission and part of the dopaminergic and serotonergic pathways and/or important for the hypothalamic-pituitary-adrenal (HPA) axis—have been suggested as related to etiology of the condition [9]. Furthermore, the most recent and so far largest genome-wide association (GWAS) meta-analysis has found evidence for chaperonin containing TCP1 subunit 5 (*CCT5*) and family with sequence similarity 173 member B (*FAM173B*) as promising candidates in pain regulation of CWP [7].

Despite evidence of a genetic basis, environmental components seem to be equally important in the pathogenesis of CWP, suggesting a possible role of gene-environment interaction in the development of the condition [3,5]. Thus, exploration of the DNA epigenetic patterns that regulate gene expression profiles may offer a new model to understand the dynamic interactions between stochastic, environmental, genetic and epigenetic factors that influence an individual`s musculoskeletal pain perception and expression. As such, specific epigenetic markers may provide quantifiable measures of lifetime environmental exposures and thus could offer a new biological framework for the cognitive-behavioral causes underlying CWP.

Recent research attempts implementing this methodology have provided promising novel insights into the etiological mechanisms underlying CWP/FM. In a genome-wide methylation study, Menzies and colleagues were able to observe significant differences in methylation patterns between 10 women with FM and 42 healthy controls, with the differentially methylated sites including brain derived neurotrophic factor (*BDNF*), N(alpha)-acetyltransferase 60, NatF catalytic subunit (*NAT15)*, or protein kinase C alpha (*PRKCA*)–all of which show biological relevance to FM [11]. While these initial results are promising, they remain unreplicated, restricted to a small sample and therefore extremely underpowered. Furthermore, and to the best of our knowledge, no studies have looked at epigenetic markers for CWP.

Given previous support for a role of methylation changes in FM-related pain, we conducted a detailed epigenome-wide analysis study (EWAS) of CWP-differentially methylated positions (DMPs) in peripheral blood DNA using the Illumina Infinium HumanMethylation 450 DNA BeadChip. Analyses were conducted on a sample of N = 281 unrelated individuals with available CWP data and in a subsample of 66 monozygotic (MZ) twins (33 twin pairs) discordant for CWP from the UK. Epigenetic studies of disease-discordant MZ twins, who are completely matched for genetics, age, sex, cohort effects, maternal influences and—to a certain extent—common environment, are considerably more powerful in detecting disease-related epigenetic differences and therefore the ideal for similar studies [12]. The 20 most significant loci from both the complete sample and the twin pairs subsample were carried forward for replication in an iindependent sample of 729 men and 756 women.

## Materials and Methods

### Participants and Study Design

The discovery sample was drawn from the TwinsUK registry, which has been shown to be representative and comparable to the general population in terms of behavior, lifestyle factors and diseases [13]. For detailed information on the twin cohort, see [14,15]. Zygosity was confirmed by genotyping [16]. Collection of CWP phenotypes and genetic/epigenetic information (from blood samples) was carried out during clinical visits and via postal questionnaires. The twin subjects were unaware of the research interests of the present study. In the end, matching phenotype and epigenome-wide data was available for a total of N = 281 subjects. All twins provided written informed consent and the study was approved by St. Thomas’ Hospital Research Ethics Committee.

The independent replication sample included 729 men and 756 women from a subsample of the KORA S4 survey, which was performed between 1999 and 2000. The KORA study includes a series of independent population-based surveys (S1-S4) recruiting participants from the region of Augsburg in Southern Germany and was initially set up as a cross-sectional survey to explore development and course of chronic diseases in a sample of adult individuals [17]. The KORA study was conducted according to the principles expressed in the Declaration of Helsinki. Written informed consent had been given by each participant. The study was reviewed and approved by the local ethical committee (Bayerische Landesärztekammer). Individuals from both the discovery and replication sample were of Caucasian background to limit biases resulting from ethnic disparities. Individuals from both the discovery and replication samples were of Northern European background to limit genetic heterogeneity.

Two different analyses were conducted in the discovery sample. First, methylation levels were compared in the full discovery sample, comprising 281 individuals. In addition, methylation status was compared in a subsample of 33 MZ twin pairs discordant for CWP. The study of epigenetics and disease using discordant MZ twins offers the opportunity to control for many potential confounders encountered in general population studies, such as differences in genetic background, early-life environmental exposure, age, gender, and cohort effects. It therefore represents a more advantaegous model and offers more power using a smaller sample to detect potential differences between disease-affected and non-affected individuals. The 20 most significant loci from both analyses (the discordant MZ subsample and the full discovery sample) were then carried forward for replication in the independent KORA sample (N = 1485).

### CWP Phenotype

Different instruments were used in the replication and discovery cohort to assess and define the main phenotype CWP. In the discovery sample, the London Fibromyalgia Epidemiology Symptom Screening Questionnaire (LFESSQ) was used to screen for self-reported CWP [18]. This 6-item questionnaire was originally designed to screen for FM in the general population and in specific patient groups and includes 4 items relating to widespread pain, and 2 items relating to fatigue. For the assessment of CWP, the four items pertaining to the “pain subscale” and asking about pain left and right of body and above and below diaphragm lasting at least 7 days in the previous 3 months were considered. In order to be classified as having CWP, participants had to respond “yes” to all four pain items with either both a right- and left-side positive response or a positive response for pain at both sides. The utility of this phenotype assessment is supported by the contribution these twins have made to previous studies, such as the genome-wide association meta-analysis conducted by Peters and colleagues [7] or the twin studies by Burri and colleagues [5].

In KORA S4, pain was assessed as part of a self-report questionnaire based on the question “To what extent did pain hinder you in your daily tasks at home and at work during the past 4 weeks?,” for which the participants could select from following answers: “no pain” (0), “not at all” (1), “slightly” (2), “moderately” (3), “quite” (4), “very” (5), “do not know.” The pain variable was examined as a dichotomous variable, identifying controls (CWPq < 3) and cases (CWPq ≥ 3).

### Illumina 450k DNA methylation analysis

#### Discovery Sample

Data and methylation analyses were conducted by common standard procedures in the discovery and the replication sample. In the discovery sample, DNA methylation analyses were performed in the R environment (http://cran.r-project.org) using raw intensity data files (IDAT files) as an input [19]. In all samples, the fraction of failed probes per sample did not exceed a quality contol cut-off (< 0.05). Multi-dimensional scaling and hierarchical clustering analyses were used to assess samples for outliers and no outliers were identified. Probes exhibiting detection p-values >0.01 were excluded from further analysis. Detection p-values predict whether the target probes signals are distinguishable from a set of background probe. For statistical analysis M-values (log2-ratios of methylated and unmethylated probes intensities) were used due to their presumed higher statistical validity [20]. Analyses of DNA methylation in 33 MZ twin pairs who screened discordant for CWP were carried out with the *RnBeads* package [21]. After removal of probes with detection p-values >0.01 in any of the samples, 483,017 probes were retained. Normalization was performed using the manufacturer-recommended scaling to internal controls as implemented in *methylumi* (method = "illumina"). Background correction was carried out with the Lumi option also as implemented in *methylumi* (bgcorr.method = "methylumi.lumi"). Paired-analysis of differential DNA methylation for the 33 MZ twin pairs was performed using paired Student's t-test. Age, array number, array position and processing batch were included as covariates in the analysis. The false discovery rate (FDR) after correction for multiple testing was set to 0.05. A priority-ranked list of differentially methylated CpGs was obtained, with rank based on a combination of statistical significance and absolute and relative effect size. The reason to select a rank approach in the discordant twin pairs analysis was to account also for effect sizes, which were the commonly very small between discordant twins.

**Fig 1:**
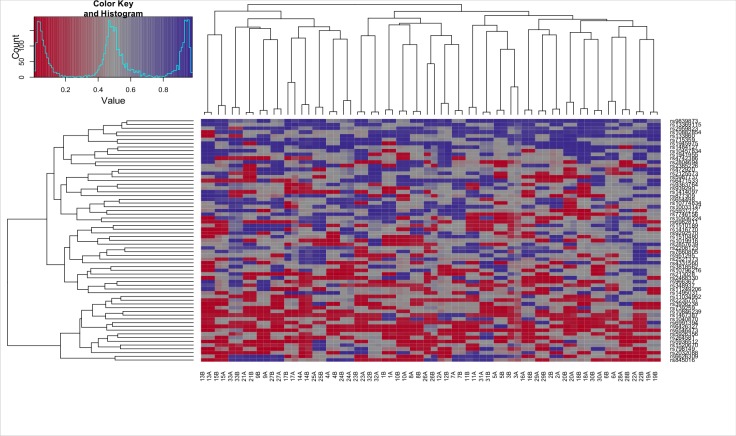
Q-Q plots in the unrelated UK sample. Before normalization (A) and after normalization (B) of the data.

DNA methylation patterns were further compared in a sample of unpaired twin individuals. One twin from each twin pair was chosen randomly, resulting in a sample of N = 281 unrelated individuals, out of which 200 screened negative and 81 screened positive for CWP. The data import, quality control and normalization steps of the *minfi* Bioconductor package were used [22,23]. Probes with detection p-values >0.01 in any of the samples were removed. Normalization and background correction were performed following the Illumina recommended procedures (bg.correct = TRUE, normalize = "controls") Q-Q plots of observed versus expected −log10 (p-values) in the unrelated CWP sample before and after normalization are shown in Fig 1. Differential methylation between the CWP positively and negatively screened individuals was estimated with the *CpGassoc* package [24]. Age, array number and array position were included as covariates in the analysis and processing batch was included as a fixed effect. The FDR (after Benjamini-Hochberg correction for multiple comparisons) was set at 0.05.

#### Replication Sample

In the replication sample, array-based DNA methylation data was also measured using the Infinium HumanMethylation450 BeadChip Array. The bisulfite conversion and genome-wide methylation assessment were performed as described in Zeilinger et al. [25]. Normalization of the methylation data was conducted following the pipeline of Lehne et al. [26] beginning with exclusion of 65 rs-probes (SNP probes) and background correction using the minfi R package. Probes were set to NA if the detection p-value was ≥0.01or if the number of beads was ≤3. Samples were excluded if the detection rate was ≤0.95. Quantile normalization, as given by Lehne et al. [26] then resulted in intensity values used to calculate the β-values. Following normalization, a site-by-site detection rate of 95% was applied to the KORA S4 cohort. To eliminate technical effects, the final methylation values for each CpG site were calculated as the residuals of a linear regression with the methylation value as response and the 20 control probe principal components (PCs) as covariates. The control probe PCs were calculated on all green and red intensities of all (non-negative, autosomal) control probes together, after background correction. The linear model for methylation (adjusted for the technical bias) and the binary CWP was estimated with the statistical software R, using age and sex as covariates.

### Database for Annotation, Visualization and Integration Discovery (DAVID) analysis

Genes to which any of the top 20 differentially methylated CpG sites were annotated were entered in the Database for Annotation, Visualization and Integration Discovery (DAVID), a high-throughput and integrated data-mining environment. Functional annotation clustering was carried out and clusters with enrichment scores > 1.3 (a previously suggested threshold) are reported [27].

## Results

The full final discovery sample of unrelated female twins (N = 281) consisted of 81 (28.8%) individuals having CWP (mean age ± SD was 62.7 ± 9.9 years). For the CWP-discordant MZ analyses, 33 full MZ twin pairs were available (mean age ± SD for the twin pairs was 61.8 ± 10.7). The final replication sample consisted of N = 1535. The overall mean age of the sample was 54.41 for men and 53.83 for women.

### Differentially methylated positions in CWP discordant MZ twins

**Fig 2:**
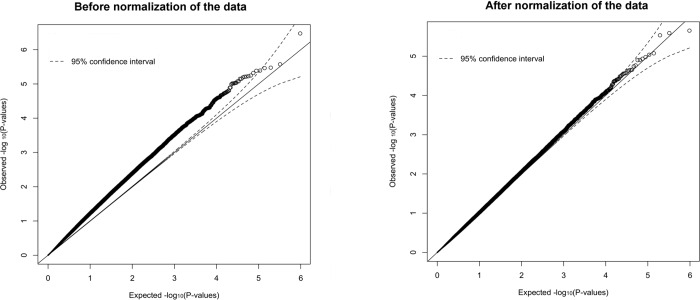
SNP Heatmap. A SNP heatmap of the 65 genotyping probes on the Illumina 450k array for the 33 CWP discordant monozygotic twin pairs.

A SNP heatmap of the 65 genotyping probes on the Illumina Infinium Human Methylation 450 BeadChip which measures single nucleotide polymorphisms, was used to assess genotype-related similarities between the samples. It showed clustering of each twin pair and no convincing additional clusters among the subjects (Fig 2). A list of the top ranked 20 DMPs between the CWP discordant twin pairs can be found in Table 1. Among the top 20 ranked DMPs we identified one mapped to monoamine oxidase B (*MAOB;* p = 0.08)–a gene previously implicated in neuropathic and postoperative pain [28,29]. Functional annotation clustering (FAC) of genes with hypermethylated CpG sites in CWP-affected MZ twins using the DAVID database further identified a cluster related to DNA binding, regulation of transcription, nucleus as well as developmental proteins (Enrichment score 1.81, genes: *BANP*, *NKX6-2*, *T*). FAC of genes with hypomethylated CpG sites in CWP-affected twins did not show any significantly enriched clusters (Table 2).

**Table 1:** Top 20 differentially methylated CpG positions in monozygotic twins discordant for CWP (N = 66) from the TwinsUK discovery sample. Included are CpG positions ranked on combination of p-values and Δβ. Within the table these positions are for consistensy arranged based on their p-values.

CpG site	Chromosome	p-value	Δβ	Gene
cg20114732	6	0.0006	-0.022	*T*
cg24847846	3	0.0021	0.04	* *
cg06782035	5	0.0023	0.0997	*March 11*
cg04246144	5	0.0025	0.024	*PCDHG*
cg25485875	10	0.0028	-0.023	*NKX6-2*
cg08270148	8	0.0034	0.024	*ERICH-AS1*
cg07518837	8	0.0037	0.025	*SPAG1*
cg03341469	1	0.0037	0.029	*YIPF1*
cg05224642	16	0.0066	-0.063	*BANP*
cg14061270	5	0.0069	0.025	* *
cg07258715	3	0.0077	0.024	*TMEM40*
cg03874092	2	0.0121	0.021	*BCL11A*
cg13749548	14	0.0125	0.063	* *
cg21210232	1	0.0126	0.03	*RNF207*
cg00324979	16	0.0137	0.04	* *
cg08629394	12	0.0158	0.021	* *
cg03548123	19	0.0179	0.042	* *
cg09357232	X	0.018	0.045	*MAOB*
cg17324941	10	0.0231	0.022	*CDC10L*
cg25173405	17	0.0256	0.028	*ITGB3*

Note: Δβ represents the difference of mean β levels of unaffected twins minus mean β levels of CWP-affected twins measured with pair-wise comparison

**Table 2:** Functional annotation cluster analysis of hypermethylated genes in the CWP-affected MZ twins from the TwinsUK sample (N = 33).

Annotation Cluster (Enrichment Score 1.81)		
Term	Genes	p-value
Developmental protein	*BANP*, *NKX6-2*, *T*	0.0016
DNA binding	*BANP*, *NKX6-2*, *T*	0.032
Regulation of transcription	*BANP*, *NKX6-2*, *T*	0.037
Nucleus	*BANP*, *NKX6-2*, *T*	0.05

### Differentially methylated sites in unrelated twin individuals

To identify DMPs in individuals with CWP compared to healthy controls in the unrelated sample, we fitted a fixed effects model regressing methylation levels at each probe on CWP status of the individuals and included age, array number, and array position as covariates and processing batch as a fixed effect. The top 20 DMPs between the two groups are presented in Table 3. These 20 DMPs included CpG islands (50%); north shores (15%); south shores (10%); as well as south shelves (5%) and sites that were not annotated into the previously noted categories (20%). Overall, the top 20 DMPs were localized around17 different genes (Table 3). The highest statistical significance between the groups was found for cg11251499, which is annotated to the 1^st^ exon/5′-UTR of RE1-silencing transcription factor (*REST)*, named also neuron-restrictive silencer factor *(NRSF*) (p-value 2.23 × 10^−6^)–a transcriptional repressor, which represses the expression of neuronal genes in non-neuronal cells [30–32]. *REST* has previously been implicated in pain perception [30]. Among the 20 most strongly associated DMRs, another previously reported biological candidate for pain—collagen type I alpha 2 chain (*COL1A2*)–could be detected. FAC of genes with hypomethylated or hypermethylated CpG sites in CWP positively screened individuals in the unrelated sample did not show significant enrichment.

**Table 3:** Top 20 differentially methylated CpG positions between unrelated individuals who screened positive (N = 81) or negative (N = 200) for CWP from the TwinsUK discovery sample.

CpGsite	Chromosome	p-value	Gene
cg11251499	4	0.00000223	*REST*
cg16116574	21	0.00000258	*WRB*
cg01832802	14	0.00000293	
cg03528037	7	0.00000845	*MDH2*
cg17658717	3	0.00000917	*CLEC3B*
cg05709655	17	0.00000105	*MRPL10*
cg01828911	8	0.0000116	*TMEM65*
cg26122815	7	0.0000125	
cg18274749	1	0.0000126	*HSPG2*
cg19439810	1	0.0000172	*LRRC41*
cg00727590	22	0.0000181	*PLAG3*
cg11721464	19	0.0000222	*DEDD2*
cg25121621	15	0.0000225	*SQRDL*
cg05368740	19	0.0000229	*HSPB6*
cg06547513	5	0.0000234	*BOD1*
cg09172260	1	0.0000262	
cg08270148	8	0.000027	*ERICH1-AS1*
cg08068547	16	0.0000275	*CLEC18C*
cg06299997	7	0.0000277	*COL1A2*
cg21533667	11	0.0000293	*TIRAP*

### Independent replication analyses of the 40 most strongly associated DMRs

The same Infinium HumanMethylation450 BeadChip Array was used for the replication of in an independent sample of n = 1485 individuals. Of these 40 DMRs, three reached EWAS significance in the indepenedent replication analyses. The three CpGs reaching significant p-values in the replication sample were malate dehydrogenase 2 (*MDH2*; p-value 0.017), tetranectin (*CLEC3B*; p-value 0.039), and heat shock protein beta-6 (*HSPB6*; p-value 0.016; Table 4). The significant associations between the previously reported biological candidates for pain identified in the discovery cohort–including *REST*, *MAOB*, *COL1A2—*could not be successfully replicated (Table 4).

**Table 4:** Replication of the 40 top hits of the TwinsUK discovery sample (using the discordant MZ subsample and the full unrelated twin invidiual sample) in the KORA sample (n = 1485).

CpGsite	Chromosome	p-value	beta	Gene
cg01832802	14	0.01320211	-0.003432303	
cg05368740	19	0.016262306	-0.010034953	*HSPB6*
cg03528037	7	0.017296535	-0.002337927	*MDH2*
cg17658717	3	0.039688442	0.009062029	*CLEC3B*
cg01828911	8	0.062226055	-0.001406351	*TMEM65*
cg08068547	16	0.110222159	-0.002210019	*CLEC18C*
cg13749548	14	0.110337895	0.035490098	
cg03874092	2	0.131432182	-0.006618897	*BCL11A*
cg26122815	7	0.162250567	-0.000894128	
cg03341469	1	0.176709411	0.005727599	*YIPF1*
cg03548123	19	0.196329334	-0.005465924	
cg20114732	6	0.210104479	0.001951687	*T*
cg08629394	12	0.338996394	0.015834355	
cg18274749	1	0.340404642	0.001334824	*HSPG2*
cg08270148	8	0.349516713	0.014808354	*ERICH-AS1*
cg08270148	8	0.349516713	0.014808354	*ERICH1-AS1*
cg19439810	1	0.361275045	-0.000813882	*LRRC41*
cg00727590	22	0.367093565	-0.001693948	*PLAG3*
cg17324941	10	0.368200397	0.005855138	*CDC10L*
cg05709655	17	0.371794037	0.000403952	*MRPL10*
cg11251499	4	0.384135807	-0.000632606	*REST*
cg11721464	19	0.410554495	0.000448264	*DEDD2*
cg06299997	7	0.420155271	-0.00498292	*COL1A2*
cg21533667	11	0.528052836	0.000200271	*TIRAP*
cg25121621	15	0.554768769	0.001318241	*SQRDL*
cg06547513	5	0.61388428	0.001285164	*BOD1*
cg09172260	1	0.625012034	-0.000801437	
cg25485875	10	0.649094944	-0.000638613	*NKX6-2*
cg07258715	3	0.76278516	0.005259776	*TMEM40*
cg16116574	21	0.789771688	-0.000457843	*WRB*
cg04246144	5	0.808636173	0.000376645	*PCDHG*
cg21210232	1	0.810668502	-0.001111336	*RNF207*
cg09357232	X	0.81928154	0.000503532	*MAOB*
cg25173405	17	0.873513415	0.000803712	*ITGB3*
cg24847846	3	0.91253349	0.000633851	
cg14061270	5	0.928871385	0.001535984	
cg05224642	16	0.940751914	0.000230206	*BANP*
cg07518837	8	0.943457128	0.000302165	*SPAG1*
cg06782035	5	0.994893721	-1.30E-05	*March 11*
cg00324979	16	0.998919474	1.67E-05	

## Discussion

Several interesting candidates could be identified in our discovery analyses, including *REST*, *MAOB*, *COL1A2*. Although none of these candidates were successfully replicated in our independent replication sample, further investigation of these candidates is warranted given previous reports of their involvement and importance in pain regulation. Although small in number, study findings in animal models and humans, for example, have repeatedly underlined the analgesic properties of reversible MAO-A inhibitors in the treatment of nociceptive and psychogenic pain [33–35]. The majority of the evidence stems from pharmacological trials investigating the therapeutic effects of MAO inhibitors on patients reporting different pain disorders. Although they are not officially approved for this indication, antidepressants–including tricyclic antidepressants (TCAs), selective serotonin reuptake inhibitors (SSRIs), and MAOIs—have been used in the treatment of FM. A study by Pillidar and colleagues [34], for example, found moclobemide—a reversible inhibitor of monoamine oxidase type A—to be an effective option in the management of perceived pain, as well as of symptoms of somatization. Similarly, a review by Tort and colleagues [36] suggested a moderate effect of MAOIs on pain and a small effect on tender points. Supporting this conclusion, a meta-analysis conducted by Häuser and colleagues found medium effect sizes for MAOIs for pain reduction [37]. Further evidence for an involvement of MAO in pain comes from genetic studies, such as the experimental study conducted by Treister et al. [38]. Using a Transmission Disequilibrium Test (TDT) on 192 individuals, the authors found significant associations between cold pain tolerance and MAO-A polymorphisms, suggesting an involvement of MAO in pain sensitivity. Similarly, the significant associations between the A/G polymorphism of the *MAOB* gene and postoperative pain intensity found by Sery et al., also point towards a potential role of *MAOB* in the perception of pain intensity [28].

Results from our larger sample of unrelated individuals also revealed a high, albeit non-significant, association between *COL1A2* and CWP. The *COL1A2* gene provides instructions for the creation of type I collagen, which is known to strengthen and support many tissues in the body (including cartilage, bone, tendon, skin, etc.). A number of previous studies have shown a link between collagen and FM. The 27 female FM patients included in a muscle biopsy study by Gronemann et al. [39], for example, showed significantly lower amount of intramuscular collagen compared to healthy controls. The authors argued that the reduced collagen may lower the threshold for muscle micro-injury and consequently resulting in non-specific signs of muscle pathology. Further exploring the distribution and amount of collagen in skin from non-tender-point areas in N = 27 female FM patients, Ribel-Madsen et al. detected differences between the amino acid composition of skin proteins, i.e., fewer amount and less density, in FM patients compared with controls [40]. Yet another study conducted on 20 FM patients even found a marginal symptomatic improvement of chronic FM symptoms by taking collagen hydrolysat–a prescription-free food supplement [41].

Functional annotation clustering of our CWP-discordant MZ results further revealed enrichment in genes with hypermethylated CpGs in the CWP-affected twins for DNA binding, regulation of transcription, nucleus as well as developmental proteins (i.e., *BANP*, *NKX6-2*). These findings are in line with a previous study by Menzies and colleagues which identified enrichment in differentially methylated sites to clusters involved in neuron differentiation, development (nervous and skeletal/organ system) and chromatin compaction in women with FM [11].

Of our 40 DMRs that were carried forward for replication, three CpGs reached significant p-values in the replication sample and included *MDH2*, *CLEC3B*, and *HSPB6*. While, to the best of our knowledge, none of them have previously been related to pain perception or expression, a recent comparative analysis of gene expression in tendinopathy found evidence of *CLEC3B* (and *COL1A2)* upregulation in tendon cells in response to cyclic strain [42]. Widespread tenderness of muscles and the areas around tendon insertions (as well as increased prevalence of tendinitis) is a common clinical presentation in patients with FM and CWP and *an involvement of CLEC3B in these* musculoskeletal and connective tissue disorders is likely [43].

### Limitations

Several study limitations have to be considered such as the relatively small sample size of the discovery sample, especially the subsample of discordant MZ twins. This might be one reason why statistical significance at FDR < 0.05 was not reached. Furthermore, comorbidities such as anxiety and depression were not taken into account, as this would have resulted in very low statistical power. Due to the relatively high mean age of our sample, extrapolation of the detected differences to other study populations is not necessarily possible. In the replication and discovery cohort, different instruments were used to assess and define the main phenotype CWP. This could be one reason why replication of the main findings fell short. However, we believe that it can equally be seen as a strength rather than a weakness that we were able to partially replicate our findings although phenotype assessment (and study population) were different. Furthermore, the different mean ages of the discovery and replication sample, as well as the fact that the cohorts were of different cultural background (UK vs Germany)–albeit homogenous in terms of ethnic background–could be other reasons why the main findings did not replicate. Finally, we used blood samples for the detection of DNA methylation patterns. It is well known that epigenetic patterns are tissue-specific, therefore the study of central and peripheral nervous system tissues would be of great interest, but are very hard to obtain in humans [44].

## Conclusion

In this large EWAS study of CWP we found evidence for the involvement of epigenetic factors. These preliminary results serve as a starting point to encourage further replication in large and independent population-based cohorts and subsequent biological investigation to elucidate possible disease mechanisms. Ultimately, understanding the key mechanisms underlying CWP may lead to new treatments and inform clinical practice and developments in psychiatric nosology.

## Supporting Information

S1 Fig Differences in DNA methylation values (Δβ) of unaffected minus CWP-affected twin for cg06782035 located at *MARCH11* (upper panel) and cg05224642 located at *BANP* (lower panel). (DOCX)

## References

[1] Wolfe F, Smythe HA, Yunus MB, Bennett RM, Bombardier C, Goldenberg DL, et al The American college of Rheumatology 1990 criteria for the classification of fibromyalgia. Report of the multicenter criteria committee. Arthritis Rheum. 1990; 33:160–72. 2306288 10.1002/art.1780330203

[2] Mansfield KE, Sim J, Jordan JL, Jordan KP. A systematic review and meta-analysis of the prevalence of chronic widespread pain in the general population. Pain. 2015; 157(1):55–64. 10.1097/j.pain.0000000000000314 PMC4711387 26270591

[3] Kato K, Sullivan PF, Evengard B, Pedersen NL. Importance of genetic influences on chronic widespread pain. Arthritis Rheum. 2006; 54:1682–1686. 10.1002/art.21798 16646040

[4] Kato K, Sullivan PF, Evengard B, Pedersen NL. Chronic widespread pain and its comorbidities: a population-based study. Arch Intern Med. 2006; 166:1649–1654. 10.1001/archinte.166.15.1649 16908799

[5] Burri A, Ogata S, Vehof J. Chronic widespread pain: clinical comorbidities and psychological correlates. Pain. 2015; 156(8):1458–64. 10.1097/j.pain.0000000000000182 25851458

[6] Wade JB, Price DD. Non-pathological factors in chronic pain: Implications for assessment and treatment In: Gatchel RJ, Weisberg NJ, eds. Personality characteristics of patients with pain. Washington: American Psychological Association; 2000:89–108.

[7] Peters MJ, Broer L, Willemen HL, Eiriksdottir G, Hocking LJ, Holliday KL. et al Genome-wide association study meta-analysis of chronic widespread pain: evidence for involvement of the 5p15.2 region. Ann Rheum Dis. 2013; 72:427–436. 10.1136/annrheumdis-2012-201742 22956598 PMC3691951

[8] Malkin I, Williams FM, Lachance G, Spector T, MacGregor AJ, Livshits G. An omics investigation into chronic widespread. Low back and common widespread pain share common genetic determinants. Pain. 2015; 156(10):1845–51. 10.1097/j.pain.0000000000000200 25915148 PMC4770329

[9] Limer KL, Nicholl BI, Thomson W, McBeth J. Exploring the genetic susceptibility of chronic widespread pain: the tender points in genetic association studies. Rheumatology (Oxford). 2008; 47:572–7 18321946 10.1093/rheumatology/ken027

[10] Livshits G, MacGregor AJ, Gieger C, Malkin I, Moayyeri A, Grallert H, et al Musculoskeletal pain reveals epiandrosterone sulfate as a potential biomarker. Ann Hum Genet. 2014; 78(5):357–66. 10.1111/ahg.12074 24962672

[11] Menzies V, Lyon DE, Archer KJ, Zhou Q, Brumelle J, Jones KH, et al Epigenetic alternations and increased frequency of micronuclei in women with fibromyalgia. Nurs Res Pract. 2013; 795784 10.1155/2013/795784 PMC3766610 24058735

[12] Bell JT, Spector TD. A twin approach to unraveling epigenetics. Trends Genet. 2011; 27(3):116–25. 10.1016/j.tig.2010.12.005 21257220 PMC3063335

[13] Andrew T, Hart DJ, Snieder H, de Lange M, Spector TD, MacGregor AJ. Are twins and singletons comparable? A study of disease-related and lifestyle characteristics in adult women. Twin Res. 2001; 4:464–477. 11780939 10.1375/1369052012803

[14] Spector TD, Williams FM. The UK Adult Twin Registry (TwinsUK). Twin Res Hum Genet. 2006; 9(6):899–906. 10.1375/183242706779462462 17254428

[15] Moayyeri A, Hammond CJ, Hart DJ, Spector TD. The UK Adult Twin Registry (TwinsUK Resource). Twin Res Hum Genet. 2013; 16(1):144–9. 10.1017/thg.2012.89 23088889 PMC3927054

[16] Von Wurmb-Schwark N, Schwark T, Christiansen L, Lorenz D, Oehmichen M. The use of different multiplex PCRs for twin zygosity determination and its application in forensic trace analysis. Legal Med. 2004; 6:125–130. 10.1016/j.legalmed.2003.12.002 15039056

[17] Holle R, Happich M, Lowel H, Wichmann HE, et al KORA–a research platform for population based health research. Gesundheitswesen. 2005; 67(suppl 1):S19–25. 16032513 10.1055/s-2005-858235

[18] White KP, Speechley M, Harth M, Ostbye T. The London Fibromyalgia Epidemiology Study: direct health care costs of fibromyalgia syndrome in London, Canada. J Rheumatol. 1999; 26(4):885–889. 10229411

[19] R_Development_Core_Team. R: A language and environment for statistical computing Vienna, Austria R Foundation for Statistical Computing 2009.

[20] Du P, Zhang X, Huang CC, Jafari N, Kibbe WA, Hou L, Lin SM. Comparison of Beta-value and M-value methods for quantifying methylation levels by microarray analysis. BMC Bioinform. 2010; 30:587. 10.1186/1471-2105-11-587 PMC3012676 21118553

[21] Assenov Y, Müller F, Lutsik P, Walter J, Lengauer T, Bock C. Comprehensive analysis of DNA methylation data with RnBeads. Nature Methods. 2014; 11:1138–1140. 10.1038/nmeth.3115 25262207 PMC4216143

[22] Gentleman RC, Carey VJ, Bates DM, et al Bioconductor: open software development for computational biology and bioinformatics. Genome Biol. 2004; 5:R80 10.1186/gb-2004-5-10-r80 15461798 PMC545600

[23] Hansen KD, Ayree M, Irizary RA, Jaffe AE, Maksimovic J, Houseman A, et al. Package “minfi”: Analyze Illumina's 450k methylation arrays. R package version 1.10.2; 2014.

[24] Barfield RT, Kilaru V, Smith AK, Conneely KN. CpGassoc: an R function for analysis of DNA methylation microarray data. Bioinform. 2012; 28:1280–1281. 10.1093/bioinformatics/bts124 PMC3577110 22451269

[25] Zeilinger S, Kuhnel B, Klopp N, Baurecht H, Kleinschmidt A, Gieger C, et al, Tobacco smoking leads to extensive genome-wide changes in DNA methylation. PLoS One. 2013; 8:e63812). 10.1371/journal.pone.0063812 23691101 PMC3656907

[26] Lehne B, Drong AW, Loh M, Zhang W, Scott WR, Tan ST, et al A coherent approach for analysis of the Illumina HumanMethylation450 BeadChip improves data quality and performance in epigenome-wide association studies. Genome Biol. 2015; 16: 37 10.1186/s13059-015-0600-x 25853392 PMC4365767

[27] Huang da W, Sherman BT, Lempicki RA. Systematic and integrative analysis of large gene lists using DAVID bioinformatics resources. Nat Protoc. 2009; 8:44–57. 10.1038/nprot.2008.211 19131956

[28] Serý O, Hrazdilová O, Didden W, Klenerová V, Staif R, Znojil V, et al The association of monoamine oxidase B functional polymorphism with postoperative pain intensity. Neuro Endocrinol Lett. 2006; 27:333–337. 16807522

[29] Villarinho JG, Oliveira SM, Silva CR, Cabreira TN, Ferreira J. Involvement of monoamine oxidase B on models of postoperative and neuropathic pain in mice. Eur J Pharmacol. 2012; 690:107–114. 10.1016/j.ejphar.2012.06.042 22771623

[30] Willis DE, Wang M, Brown E, Fones L, Cave JW. Selective repression of gene expression in neuropathic pain by the Neuron-Restrictive Silencing Factor/Repressor Element-1 Silencing Transcription (NRSF/REST). Neurosci Lett. 2015;pii: S0304-3940(15):30293–7. 10.1016/j.neulet.2015.12.003 PMC4899316 26679228

[31] Uchida H, Ma L, Ueda H. Epigenetic gene silencing underlies C-fiber dysfunctions in neuropathic pain. J Neurosci. 2010; 30:4806–4814. 10.1523/JNEUROSCI.5541-09.2010 20357131 PMC6632306

[32] Lu C, Shi L, Zhang J, et al Neuron-restrictive silencer factor in periaqueductal gray contributes to remifentanil-induced postoperative hyperalgesia via repression of the mu-opioid receptor. J Neurol Sci. 2015; 352:48–52. 10.1016/j.jns.2015.03.018 25819118

[33] Coquoz D, Porchet HC, Dayer P. Central analgesic effects of desipramine, fluvoxamine, and moclobemide after single oral dosing: a study in healthy volunteers. Clin Pharmacol Ther. 1993; 54:339–344 8375130 10.1038/clpt.1993.156

[34] Pirildar S, Sezgin U, Elbi H, Uyar M, Zileli B. A preliminary open-label study of moclobemide treatment of pain disorder. Psychopharmacol. Bull 2003; 37:127–134. 14608245

[35] Schreiber V, Getslev A, Weizman GG. Pick. The antinociceptive effect of moclobemide in mice is mediated by noradrenergic pathways. Neurosci Lett. 1998; 253:183–186 9792241 10.1016/s0304-3940(98)00638-7

[36] Tort S, Urrútia G, Nishishinya MB, Walitt B. Monoamine oxidase inhibitors (MAOIs) for fibromyalgia syndrome. Cochrane Database Syst Rev. 2012 8; 4:CD009807 10.1002/14651858.CD009807 PMC11729144 22513976

[37] Häuser W, Bernardy K, Uçeyler N, Sommer C. Treatment of fibromyalgia syndrome with antidepressants: a meta-analysis. JAMA. 2009; 301(2):198–209. 10.1001/jama.2008.944 19141768

[38] Treister R, Pud D, Ebstein RP, Laiba E, Gershon E, Haddad M, et al Associations between polymorphisms in dopamine neurotransmitter pathway genes and pain response in healthy humans. Pain. 2009; 147(1–3):187–93. 10.1016/j.pain.2009.09.001 19796878

[39] Gronemann ST, Ribel-Madsen S, Bartels EM, Danneskiold-Samsoe B, Bliddal H. Collagen and muscle pathology in fibromyalgia patients. Rheumatology (Oxford). 2004; 43(1):27–31. 12867573 10.1093/rheumatology/keg452

[40] Ribel-Madsen S, Gronemann ST, Bartels EM, et al Collagen structure in skin from fibromyalgia patients. Int J Tissue React. 2005; 27(3):75–82. 16372472

[41] Olson GB, Savage S, Olson J. The effects of collagen hydrolysat on symptoms of chronic fibromyalgia and temporomandibular joint pain. Cranio. 2000; 18(2):135–41. 11202824 10.1080/08869634.2000.11746125

[42] Choi WJ, Park MS, Park KH, Courneya JP, Cho JS, Schon LC, Lee JW. Comparative analysis of gene expression in normal and degenerative human tendon cells: effects of cyclic strain. Foot Ankle Int. 2014 10;35(10):1045–56. 10.1177/1071100714540885 24958764

[43] Genç H, Saracoğlu M, Duyur B, Erdem HR. The role of tendinitis in fibromyalgia syndrome. Yonsei Med J. 2003;44(4):619–22. 10.3349/ymj.2003.44.4.619 12950117

[44] Szyf M, Bick J. DNA methylation: a mechanism for embedding early life experiences in the genome. Child Dev. 2013; 84(1):49–57. 10.1111/j.1467-8624.2012.01793.x 22880724 PMC4039199

