# Supplementary material for: Are Epigenetic Factors Implicated in Chronic Widespread Pain?
Source: PLoS One. 2016 Nov 10;11(11):e0165548. doi: 10.1371/journal.pone.0165548 (PMC5104434; doi:10.1371/journal.pone.0165548)
Supplement: S1 Fig — (DOCX) [file pone.0165548.s001.docx]

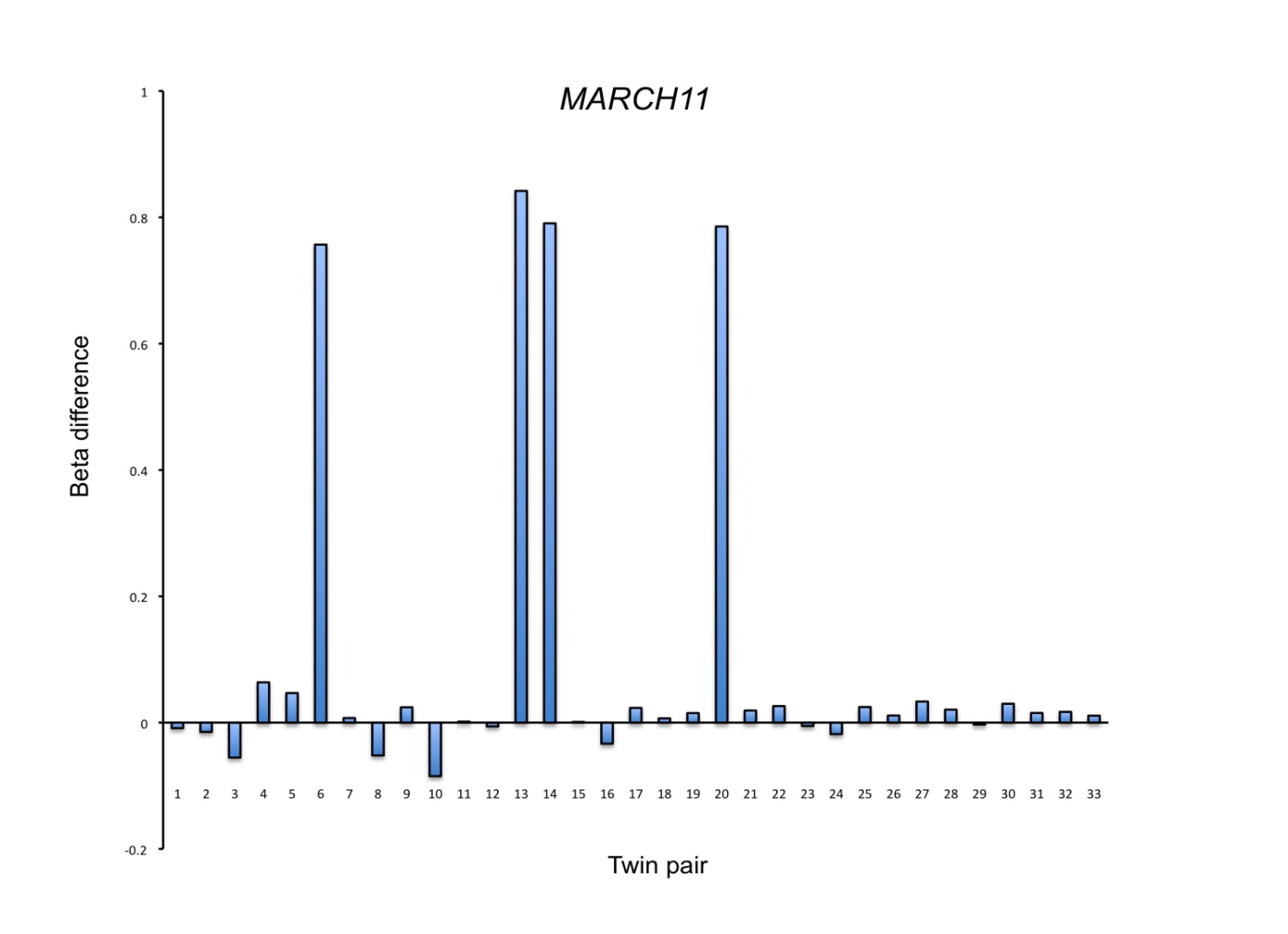


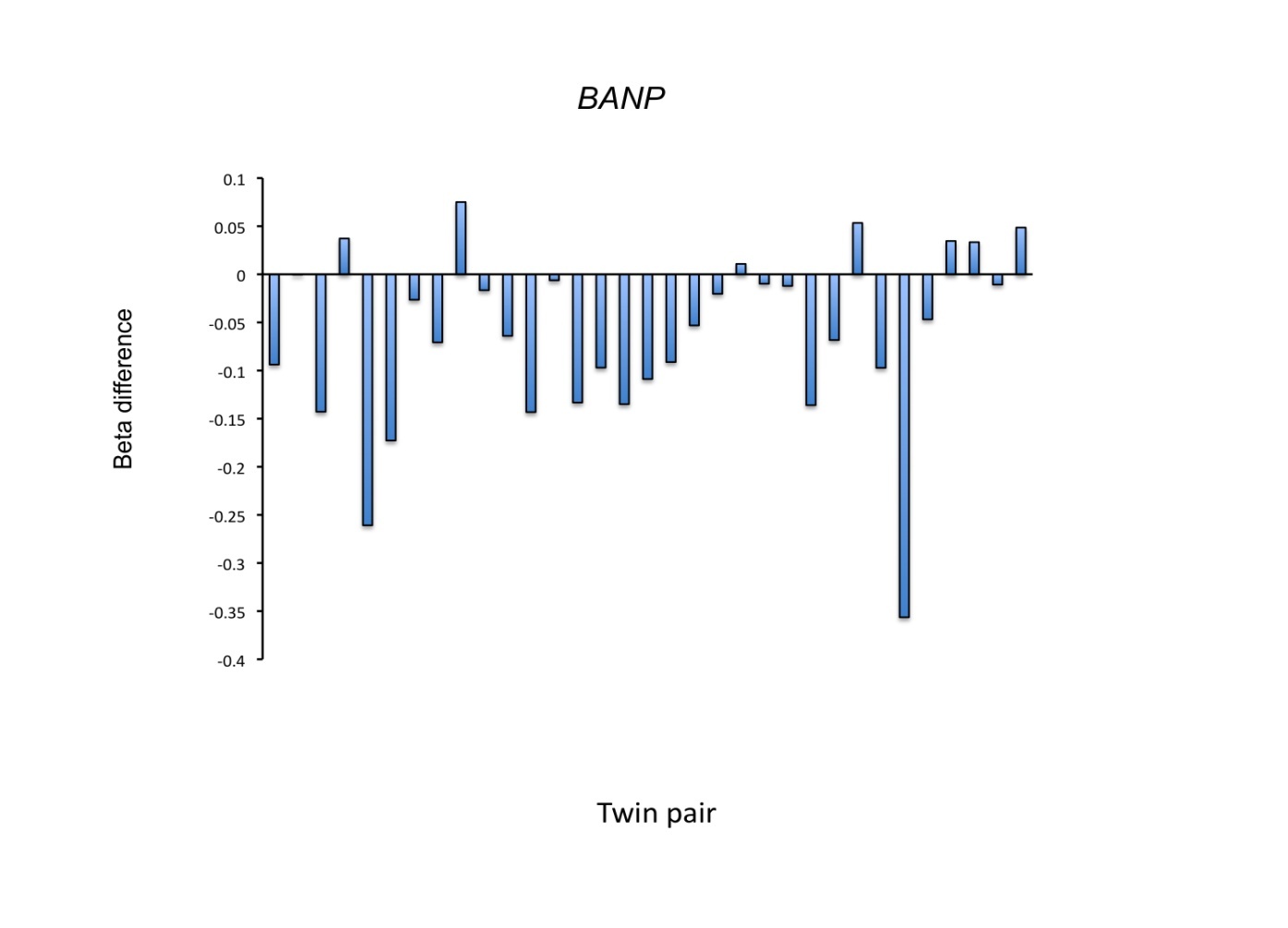


**Supplementary Figure S1.** Differences in DNA methylation values (∆β) of unaffected minus CWP-affected twin for cg06782035 located at *MARCH11* (upper panel) and cg05224642 located at *BANP* (lower panel).
